# CAPABLE: APPLYING AN EVIDENCE-BASED MODEL OF CARE FOR OLDER ADULTS WITH HIV

**DOI:** 10.1093/geroni/igad104.0381

**Published:** 2023-12-21

**Authors:** Jamie Smith, Jason Farley, Kelly Lowensen, Kennedy McDaniel, Sarah Szanton, Katherine Ornstein

**Affiliations:** Johns Hopkins University, Baltimore, Maryland, United States; Johns Hopkins University, Baltimore, Maryland, United States; Johns Hopkins University, Baltimore, Maryland, United States; Johns Hopkins School of Nursing, Baltimore, Maryland, United States; Johns Hopkins University, Baltimore, Maryland, United States; Johns Hopkins University, Baltimore, Maryland, United States

## Abstract

In the United States, more than half of people living with HIV (OWH) are at least 50 years. Older PWH experience increased comorbidities, earlier onset of geriatric syndromes, and functional decline. These health consequences intersect with complex social and environmental factors, further complicating aging with HIV. Yet initiatives focused on successful aging do not consider the unique needs of older PHWs. Models of care tailored to the individual that addresses both intrinsic (e.g., chronic infections, stress, and nutrition) and extrinsic (socioeconomic status, housing instability, food insecurity, and threats to safety) factors unique to older PWH are needed to improve health and functional outcomes. Community Aging in Place, Advancing Better Living for Elders (CAPABLE), an internationally recognized model of care, is a person-directed program that has been successful in supporting aging in place by reducing functional decline and disability among older adults. Over several months, the participant, occupational therapist, registered nurse, and handy worker collaborate on solutions to improve the participant’s bio-psych-functional capacity to function at home. In multiple trials, CAPABLE has demonstrated improvements in independence, safety, self-efficacy, and depression. CAPABLE is associated with healthcare cost reductions, including over $30,000 in Medicare and Medicaid savings. CAPABLE has not been adapted to older PWH, despite being well-positioned to address intrinsic and extrinsic factors affecting adherence, viral suppression, and other health outcomes. This presentation will discuss the first-ever translation of CAPABLE to a cohort of older PWH using human-centered design strategies and discuss initial feasibility trial of older PWH at Johns Hopkins.

